# PLACE AND MEANING OF HOME: RESEARCH IN A VIRTUAL, PHOTOVOICE LENS

**DOI:** 10.1093/geroni/igac059.976

**Published:** 2022-12-20

**Authors:** Joyce Weil

**Affiliations:** University of Northern Colorado, Greeley, Colorado, United States

## Abstract

Photovoice is built upon the principle of empowerment. With the increased use of Smartphones, photovoice provides a more readily available qualitative option for co-research with older adults. This presentation provides examples from the current study illustrating the process of creating a project that uses photovoice to see how older adults construct the meaning of home and place. While the benefits of this design are many, some issues do arise during implementation, such as: creating study protocols to address Institutional Review Board (IRB) concerns; working with organizations to ensure that the design is truly co-created; and navigating and conducting photovoice in a completely virtual arena. Examples will also be drawn from technical training received as part of LeadingAge LTSS Center @UMass Boston and Collective Insight’s Aging Centered Outcomes Research Learning Collaborative, as well as the Healthier Black Elders Center, which is affiliated with the University of Michigan and Wayne State University.

